# Ambulance crew‐initiated non‐conveyance in the Helsinki EMS system—A retrospective cohort study

**DOI:** 10.1111/aas.14049

**Published:** 2022-02-28

**Authors:** Kari Heinonen, Tuukka Puolakka, Heli Salmi, James Boyd, Mia Laiho, Kari Porthan, Heini Harve‐Rytsälä, Markku Kuisma

**Affiliations:** ^1^ Department of Emergency Medicine & Services Helsinki University Hospital and University of Helsinki Helsinki Finland; ^2^ Department of Anesthesiology & Intensive Care Medicine Helsinki University Hospital and University of Helsinki Helsinki Finland; ^3^ 3852 Parliament of Finland Helsinki Finland; ^4^ Helsinki City Rescue Department Helsinki Finland

**Keywords:** emergency medical services, mortality, non‐conveyance, patient transport

## Abstract

**Background:**

Ambulance patients are usually transported to the hospital in the emergency medical service (EMS) system. The aim of this study was to describe the non‐conveyance practice in the Helsinki EMS system and to report mortality following non‐conveyance decisions.

**Methods:**

All prehospital patients ≥16 years attended by the EMS but not transported to a hospital during 2013–2017 were included in the study. EMS mission‐ and patient‐related factors were collected and examined in relation to patient death within 30 days of the EMS non‐conveyance decision.

**Results:**

The EMS performed 324,207 missions with a patient during the study period. The patient was not transported in 95,909 (29.6%) missions; 72,233 missions met the study criteria. The patient mean age (standard deviation) was 59.5 (22.5) years; 55.5% of patients were female. The most common dispatch codes were malaise (15.0%), suspected decline in vital signs (14.0%), and falling over (12.9%). A total of 960 (1.3%) patients died within 30 days after the non‐conveyance decision. Multivariate logistic regression analysis revealed that mortality was associated with the patient's inability to walk (odds ratio 3.19, 95% confidence interval 2.67–3.80), ambulance dispatch due to shortness of breath (2.73, 2.27–3.27), decreased level of consciousness (2.72, 1.75–4.10), decreased blood oxygen saturation (2.64, 2.27–3.06), and abnormal systolic blood pressure (2.48, 1.79–3.37).

**Conclusion:**

One‐third of EMS missions did not result in patient transport to the hospital. Thirty‐day mortality was 1.3%. Abnormalities in multiple respiratory‐related vital signs were associated with an increased likelihood of death within 30 days.

**Editorial Comment**

The decision in the field for an ambulance crew to transport a patient to the hospital or not can be challenging. This report from a large national capital prehospital system presents expererience with this, including follow‐up at one month.

## 1 INTRODUCTION

The traditional purpose of emergency medical services (EMS) is to respond to acute medical emergencies and trauma and to provide emergency care and ambulance transportation. However, there has been an emerging trend to engage the EMS system at a lower threshold; this phenomenon is associated with an ageing population, limited access to primary health care, poor health literacy, and easy access to an emergency phone number (112, 911, or 999).^1^, ^2^, ^3^ As much as half of all urgent ambulance transport to emergency departments (ED) may be medically unjustified.^4^ In recent years, the increasing patient flow and ED overcrowding has become a problem in the hospital system.^5^, ^6^

In the EMS system, the term non‐conveyance means that the patient is not transported to a hospital but is discharged on‐scene after successful evaluation or treatment. Previously, the possibility of non‐conveyance was limited to cases of patient refusal. Many systems have recently started to allow emerging non‐conveyance practices.^7^, ^8^, ^9^, ^10^ However, it is unclear which patient groups are suitable for discharge on‐scene and whether non‐conveyance poses a threat to patient safety.^11^

The aim of this study was to describe the ambulance crew‐initiated non‐conveyance practice of the Helsinki EMS system, to report 30‐day mortality in an urban EMS system with protocols enabling ambulance crew‐initiated non‐conveyance, and to describe factors related to non‐conveyance decisions.

## 2 METHODS

This was a 5‐year retrospective cohort study combining prehospital patient reports and mortality data. The study was approved by the Hospital District of Helsinki University Hospital (HUS) review board (HUS 278/2018) according to Finnish legislation. As this was a register‐based study, separate approval from the Ethics Committee and patient informed consent were not required. The study was designed in accordance with the STROBE statement.^12^

### 2.1 Study setting

HUS organises the EMS in the capital city of Helsinki and the surrounding metropolitan area. Helsinki has a population of 630,000 and a geographical area of 214.25 km^2^.^13^

All emergency phone calls to the uniform emergency number 112 are handled by the National Emergency Response Centre. All missions are given a dispatch code consisting of the prespecified reason for dispatch and a letter indicating the triage level (A to D), with the two highest levels attended by the closest unit with lights and sirens. If the dispatcher evaluates that no ambulance is required, the caller may be advised to contact primary healthcare providers or call a helpline providing non‐urgent medical advice. The three‐tiered EMS system consists of basic life support (BLS) and advanced life support (ALS) ambulances supported by a medical supervisor unit with a senior paramedic and a physician‐staffed mobile intensive care unit, which also provides online telecommunication support for ambulances in addition to being called on‐scene when necessary.

A uniform electronic patient reporting system (EPR, Merlot Medi^®^, CGI Inc., Canada), is used to record all EMS missions. Vital signs are measured using LIFEPAK series monitor‐defibrillators (Physio Control Inc, Redmond, WA, USA) and are transmitted automatically to the EPR. Tympanic temperature, respiratory rate, and level of consciousness (GCS) are entered manually by the ambulance crew. The EPR software automatically calculates the National Early Warning Score (NEWS).^14^ A single measured vital sign is given a score from 0 (normal) to 3 (highly abnormal) to calculate a total risk score out of a maximum of 21 NEWS points. In the NEWS score, 0–4 points are considered low risk, 5–6 medium risk, and >6 is considered high risk. Patient history of prior EMS missions and ECGs can be viewed from the EPR on‐scene. The EMS physician can follow all data stored in the EPR and hospital records in real time, which facilitates consultations and supervision.

### 2.2 Non‐conveyance practice in Helsinki

The practice of ambulance crew‐initiated non‐conveyance in the Helsinki EMS system dates back to the 1980s, from where it has evolved from single ‘ad hoc’ decisions to a uniform decision‐making model regulated by guidelines and supervised by EMS physicians. Most decisions concerning patient care and conveyance were and are still made independently by the ambulance crews. The patient must fulfil specific criteria stated in the local non‐conveyance protocol (see Additional File 1). In most cases, this means that the symptoms that led to the emergency phone call have passed or that the aetiology is clear and does not require further assessment in the ED. Vital signs should be close to normal or have a clear reason for their abnormality (e.g. tachycardia following a panic attack). In some cases, the patient has a specific problem that can be evaluated and treated satisfactorily by the ambulance crew (e.g. hypoglycaemia treated with intravenous glucose). In all cases of non‐conveyance, the ambulance personnel is required to answer a standardised checklist (Additional File 1). If the decision of non‐conveyance is not clear, the EMS personnel must consult the EMS physician, who makes the final decision between transport and non‐conveyance. The physician must be consulted when a patient refuses transportation that would clearly be in the patient's best interests, or when non‐conveyance is seen as the best option for a critically ill patient (e.g. terminally ill patients who wish to die at home). A system of internal quality control is used to assess that protocols have been followed accordingly. All patients are examined on‐scene by the EMS personnel and decisions are based on patient assessment, vital signs, patient history, and data available in previous EMS records. The reason for non‐conveyance is recorded in all cases. A specific ICD‐10 diagnosis is recorded only in cases in which the EMS physician is on‐scene to examine the patient. The EMS provider receives compensation for the EMS mission regardless of the patient's transport, which removes any financial incentives related to decisions of non‐conveyance.

### 2.3 Data collection and statistical analysis

This study focused on EMS missions in which the decision of non‐conveyance was made by the EMS personnel. All EMS missions from a 5‐year period from 1 January 2013 to 31 December 2017 that did not result in transport were collected from the EPR system. Exclusion criteria included patient age < 16 years, patients dying on‐scene, patients not found or refused transport, and patients with incomplete personal identification codes. The final measurements prior to non‐conveyance were used to calculate the NEWS score registered from each patient. For each single vital sign, we considered a score of 0 to 1 as ‘normal’ and 2 to 3 as ‘abnormal’. The EMS personnel do not have to measure a full set of vital signs in patients who are clearly well. Therefore, we interpreted missing values as normal. Because no patient was left on‐scene with supplemental oxygen, these points were excluded. Mortality data were obtained from Statistics Finland.^15^ The primary outcome measure was death within 30 days of the EMS mission. Mortality was compared to both patient‐ and EMS mission‐related data.

All data were collected in Microsoft Excel format. The latest versions of IBM SPSS Statistics (IBM, NY, USA) and R (R Foundation for Statistical Computing, Vienna, Austria) were used to conduct the statistical analyses. Pearson χ^2^ and Student's *t*‐test were used to identify factors associated with 30‐day mortality. A further multivariate model analysis using logistic regression was conducted. EMS missions to nursing homes were analysed separately from other EMS missions, due to likely multiple comorbidities and patients not being independent in their daily life functions. The variables in the model were selected based on clinical judgement and statistical significance in the univariate analysis. The results are shown with forest plots made with the ggplot2 package. The mortality risks shown in the multivariate model are further illustrated using relative risk tables to show the effect of the different risk factor combinations.

## 3 RESULTS

**FIGURE 1:**
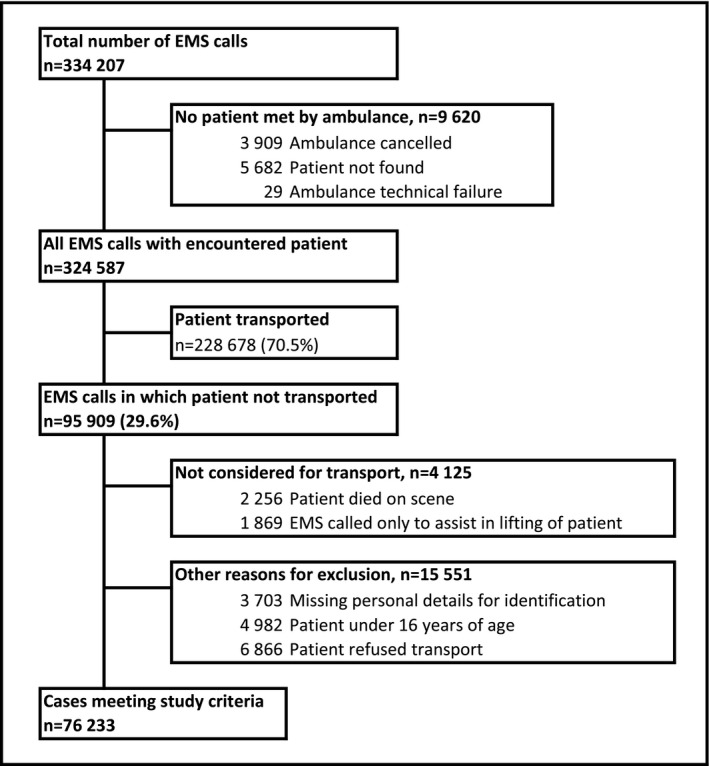
Description of the study sample

The EMS patient was not transported in 95,909 EMS missions during the study period. Of these missions, 76,233 met the study criteria (Figure 1). The sample is described in detail in Table 1. The emergency phone calls were most often made by the patients themselves. Most EMS missions (60,003, 78.7%) were attended by BLS‐level ambulance crews. Heart rate, non‐invasive blood pressure, and blood oxygen saturation were the most commonly measured vital signs; these were measured for 66,484 patients (87.2%). The EMS physician was consulted in 9176 (12.0%) missions. The most common reason for a non‐conveyance decision made by the EMS personnel in 67,303 patients (88.3%) was that ambulance transportation or emergency care was not required and no specific treatments or medications were given at the scene (Table 1).

**TABLE 1:** Description of emergency medical services (EMS) calls

Variable	All EMS calls (*n* = 76,233)
Patient	
Female	55.5%
Age, years	59.5 (22.5)
Number of prior EMS calls during past 365 days	1 (0–3)
Living in a nursing home	4.7%
Death within 30 days after non‐conveyance	1.3%
Dispatch	
Person who made EMS call	
Patient	35.1%
Family member	21.0%
Bystander	24.3%
Medical professional	9.4%
Police or rescue department responder	1.3%
Final dispatch code	
Malaise	15.0%
Suspected decline in vital functions	14.0%
Falling over	12.9%
Chest pain	8.9%
Musculoskeletal pain	6.3%
Shortness of breath	5.8%
Mental illness	2.7%
Other	34.4%
Dispatch triage level	
A	2.3%
B	19.4%
C	49.3%
D	28.8%
EMS call	
Highest level of non‐physician unit on scene	
Basic life support	78.2%
Advanced life support	21.2%
First responder unit (fire engine) only	0.1%
EMS physician on‐scene	0.9%
EMS physician consulted	12.0%
On‐scene time, minutes	21.0 (16–29)
Measurements taken	
Heart rate	95.0%
Blood pressure	94.8%
Blood oxygen saturation	87.6%
Body temperature	69.1%
Level of consciousness	63.0%
Respiratory rate	47.1%
Breath alcohol content	30.9%
Treatment given	
Intravenous fluids	2.2%
Any medication	0.9%
Supplemental oxygen	0.1%
Reason for non‐conveyance	
No transport required	88.3%
Treated on the scene	5.0%
Other means of transport	4.9%
Taken into police custody	1.5%
Other assistance requested	0.3%

Note

Data are presented as mean (standard deviation), median (interquartile range), or percentage.

**FIGURE 2:**
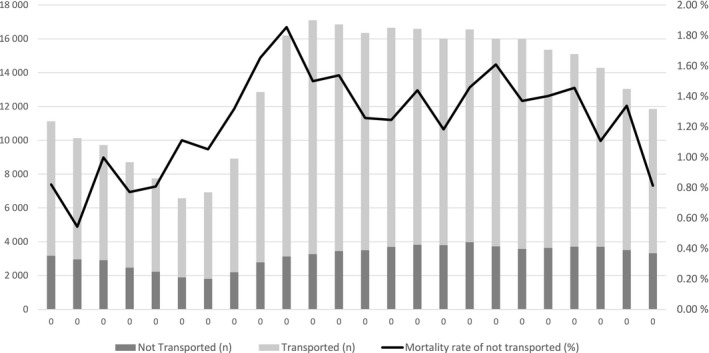
Number of EMS missions and 30‐day mortality rate of non‐transported patients by time of dispatch

In 960 missions, the patient died within 30 days of the non‐conveyance decision (overall mortality 1.3%). Only 97 (0.13%) patients died during the first 24 h and 152 (0.20%) during the first 72 h after the non‐conveyance decision. Only 128 (13.3%) of all deaths occurred among patients <60 years. Table 2 compares the patients who were alive and those who died 30 days after the non‐conveyance decision. More than one‐fifth of the deceased patients (208, 21.7%) were identified as nursing home residents. It was also more common that these patients did not make the emergency phone call by themselves but relied on outside help (Table 2). The dispatch codes were similar in both groups, with shortness of breath overrepresented in the deceased population. The highest mortality was observed in patients with two to three prior missions during the past year. A prior EMS mission within the previous 48 h had no significant effect on mortality. The mortality rate was the highest for EMS missions dispatched between 9.00 AM and 10.00 AM and lowest for missions during the night hours from 11.00 PM to 5.00 AM (Figure 2). During the night hours, 60.1% of the patients were <60 years; the corresponding value was 42.0% during other hours. The most common causes of death were chronic diseases, such as neoplasms (194, 20.2%), respiratory tract infections (129, 13.4%), and ischaemic heart disease (127, 13.2%).

**TABLE 2:** Comparison of risk factors between patients alive and deceased at 30 days after non‐conveyance decision

	Death within 30 days (*n* = 960)	Alive (*n* = 75,273)	Relative Risk (95% CI)	*p*‐value
Patient background				
Age, years	77.1 (14.5)	59.3 (22.5)		<.001
Female	51.1%	55.6%	0.84 (0.74–0.95)	.006
Previously healthy	2.9%	16.8%	0.15 (0.11–0.22)	<.001
Living in a nursing home	21.7%	4.5%	5.56 (4.79–6.46)	<.001
Unable to walk	19.9%	3.9%	5.75 (4.93–6.71)	<.001
DNAR order	8.8%	0.3%	22.89 (18.84–27.82)	<.001
Person who made EMS call				
Patient	16.9%	35.4%	0.38 (0.32–0.44)	<.001
Family member	27.5%	20.9%	1.43 (1.24–1.65)	<.001
Bystander	13.9%	24.4%	0.51 (0.42–0.61)	<.001
Medical professional	31.6%	9.1%	4.47 (3.91–5.11)	<.001
Unknown	8.1%	8.3%	1.03 (0.82–1.23)	.809
Dispatch				
Triage level A or B	23.9%	21.7%	1.12 (0.97–1.30)	.102
Malaise	21.5%	15.0%	1.54 (1.32–1.80)	<.001
Suspected decline in vital functions	8.1%	14.0%	0.54 (0.43–0.69)	<.001
Falling over	15.9%	12.9%	1.28 (1.08–1.52)	.005
Chest pain	5.3%	8.9%	0.58 (0.44–0.76)	<.001
Musculoskeletal pain	5.3%	6.3%	0.84 (0.63–1.11)	.206
Shortness of breath	17.9%	5.7%	3.54 (3.01–4.16)	<.001
Convulsion	1.4%	2.8%	0.49 (0.28–0.84)	.008
Mental illness	1.3%	2.7%	0.46 (0.26–0.81)	.006
EMS call				
ALS‐trained crew on the scene	19.4%	21.3%	1.12 (0.96–1.32)	.157
EMS physician consulted	19.7%	11.9%	1.79 (1.53–2.10)	<.001
EMS physician on‐scene	2.8%	0.8%	3.31 (2.33–4.81)	<.001
Abnormal measurements				
Systolic blood pressure	5.1%	1.4%	3.74 (2.83–4.96)	<.001
Heart rate	9.1%	8.3%	1.10 (0.89–1.37)	.376
Blood oxygen saturation	28.5%	8.1%	4.35 (3.79–5.00)	<.001
Respiratory rate	5.2%	1.5%	3.54 (2.68–4.68)	<.001
Level of consciousness	3.1%	0.4%	6.99 (4.94–9.91)	<.001
Body temperature	1.8%	1.6%	1.11 (0.69–1.79)	.657
Breath alcohol content	10.6%	23.0%	0.40 (0.33–0.49)	<.001
Treatment given				
Any medication	1.7%	0.9%	1.95 (1.20–3.17)	.007
Intravenous fluids	2.1%	2.2%	0.95 (0.61–1.48)	.823
Supplemental oxygen	1.3%	0.1%	10.25 (6.02–17.46)	<.001
Number of abnormal vital signs				
0 abnormal vitals	59.4%	80.8%	0.35 (0.31–0.40)	<.001
1 abnormal vital	31.1%	17.3%	2.13 (1.86–2.44)	<.001
2 or more abnormal vitals	9.5%	1.9%	5.09 (4.13–6.28)	<.001
National Early Warning Score				
0	21.8%	43.7%	0.35 (0.30–0.41)	<.001
1–2	46.2%	44.2%	1.05 (0.92–1.19)	.492
3–4	23.0%	10.4%	2.46 (2.12–2.86)	<.001
5–6	7.7%	1.6%	4.86 (3.84–6.13)	<.001
>7	1.4%	0.1%	10.25 (6.14–17.09)	<.001
Patient's total number of EMS calls during the previous 365 days				
0	29.0%	47.7%	0.45 (0.39–0.51)	<.001
1	22.6%	16.3%	1.50 (1.29–1.74)	<.001
2–3	23.8%	14.6%	1.80 (1.56–2.09)	<.001
4–9	16.4%	12.2%	1.40 (1.18–1.66)	<.001
10–19	5.7%	4.8%	1.20 (0.91–1.57)	.194
≥20	2.6%	4.4%	0.58 (0.39–0.86)	.006
Reason for non‐conveyance				
No transport needed	89.1%	88.3%	1.08 (0.88–1.32)	.451
Other means of transport	4.8%	4.9%	0.97 (0.72–1.30)	.845
Treated on scene	4.1%	5.0%	0.81 (0.59–1.11)	.184
Taken into police custody	0.8%	1.5%	0.57 (0.28–1.14)	.104
Other assistance requested	1.3%	0.3%	3.71 (2.13–6.48)	<.001

Note

Data are presented as percentage, mean (standard deviation), or relative risk (95% confidence interval). Last measured value for each vital parameter was registered. National early warning score of 2–3 points was considered abnormal.

Abbreviations: ALS, advanced life support; EMS, emergency medical service.

The association of abnormal vital signs with increased mortality was most clearly seen in abnormal levels of consciousness, blood oxygen saturation, and systolic blood pressure. Abnormal heart rate or abnormal body temperature was not associated with mortality (Table 2). However, 457 (47.6%) of the deceased patients had 0 or 1 NEWS points during the EMS mission.

**FIGURE 3:**
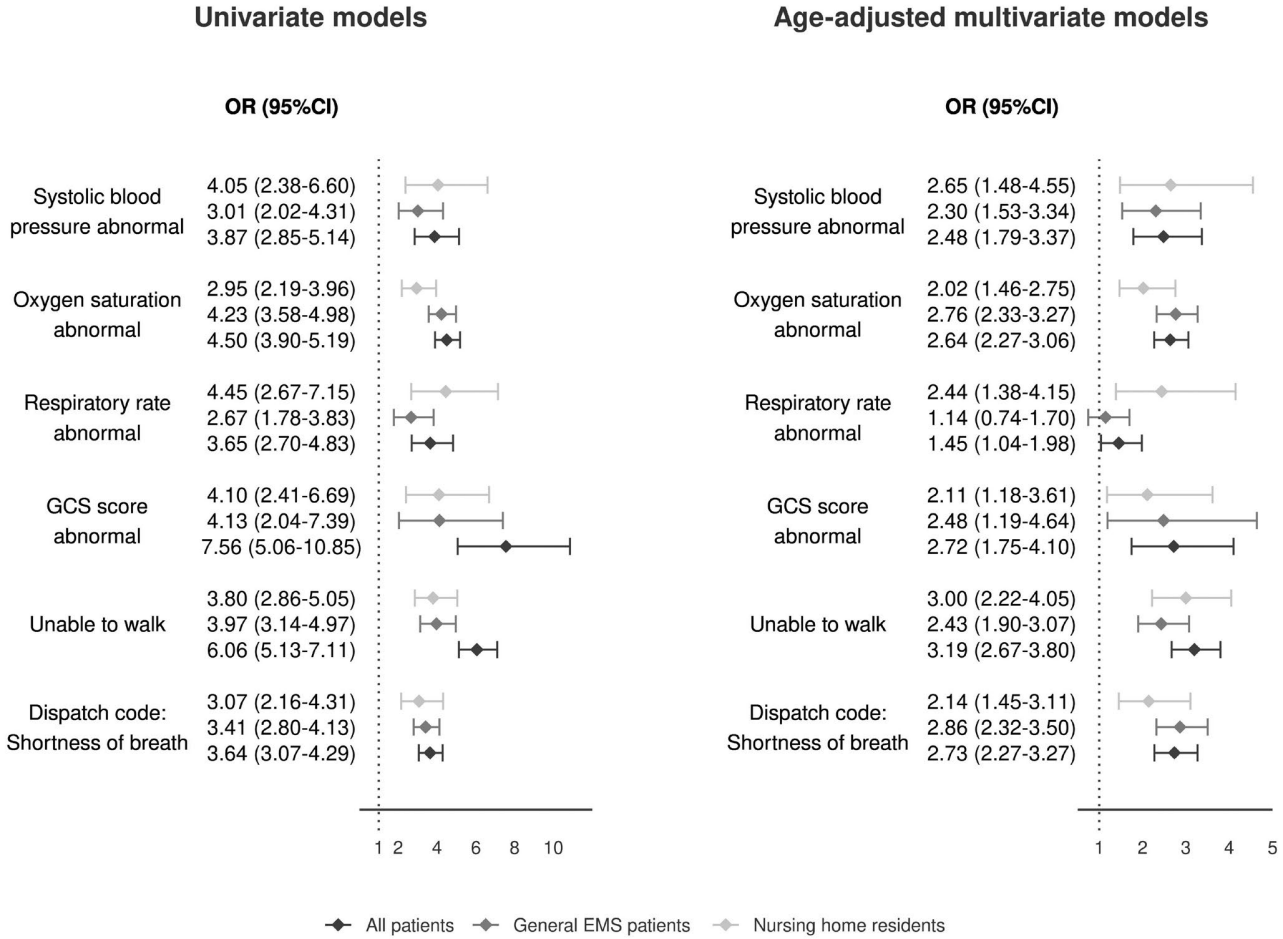
Univariate and multivariate models of risk factors with highest 30‐day mortality factored by nursing home occupancy. The ORs (95% confidence intervals) for log(age) were the following: all patients 14.26 (10.73–19.20), general EMS patients 11.62 (4.31–34.55), nursing home residents 11.88 (8.84–16.19). A vital sign was considered abnormal if the last measured value yielded 2 or 3 points on the National Early Warning Score. EMS, Emergency Medical Service; GCS, Glasgow Coma Scale

The risk factors with the strongest association with 30‐day mortality were analysed using logistic regression. The inability to walk (odds ratio [OR] 3.00, 95% confidence interval [CI] 2.22–4.05) and abnormal blood pressure (OR 2.65, 95% CI 1.48–4.55) were the highest ORs among the nursing home population. A combination of these two risk factors had a relative risk of 7.2 when compared to nursing home patients with no risk factors. Similarly, in the general EMS patient population, shortness of breath (OR 2.86, 95% CI 1.90–3.50) and low blood oxygen saturation (OR 2.76, 95% CI 2.33–3.27) combined had a relative risk of 7.6. The results for the multivariate and age‐adjusted models are presented in Figure 3, and a full analysis of multiple comorbidities is shown in Additional Table S1.

## 4 DISCUSSION

To the best of our knowledge, this is the largest study describing a universal, ambulance crew‐initiated, prehospital non‐conveyance practice. Nearly a third of all EMS missions resulted in non‐conveyance after patient evaluation. Direct comparison of EMS practice is difficult since EMS systems, operational protocols, and national legislations vary. Previous studies of prehospital non‐conveyance practices have been mostly limited to small, often disease‐ or symptom‐specific patient groups. The non‐conveyance rate in these studies has varied from 3.7% to 93.7%, and the reported mortality at 24 and 72 h has been 0.2 to 3.5% and 0.3 to 6.1%, respectively.^16^, ^17^, ^18^, ^19^ In this large dataset from a city‐based EMS system, the non‐conveyance rate was in the midrange when compared with previous studies but was lower than in previous reports from Finland, which were focused on rural settings.^20^, ^21^

It is noteworthy that most dispatches were due to non‐specific complaints, such as malaise or suspected deterioration of vital signs. This describes the difficulty of evaluating mild, chronic, and complicated medical problems during the emergency‐oriented dispatch process. In many countries, telephone services intended for the assessment of non‐urgent medical problems are available. One of the most advanced of these systems is located in Copenhagen, Denmark, where a telephone contact to a medical professional is used as an alternative for an ambulance.^22^ However, dyspnoea was strongly associated with increased mortality already during emergency phone call processing and should be considered as a high‐risk symptom. This was further emphasised by the fact that both decreased blood oxygen saturation and abnormal respiratory rate were also associated with increased mortality in a multivariate regression model.

This study was not designed to compare mortality between conveyed and non‐conveyed patients. Instead, we described our non‐conveyance practice and its outcomes. As there are previous reports on the outcomes of non‐conveyed patients, we considered comparing our mortality rates to these reports, keeping in mind that the EMS systems and studied populations are different. Despite a high non‐conveyance rate, we note that the short‐term mortality in our non‐conveyed patients was in the lower range of all previous reports on non‐conveyance.^16^, ^17^, ^18^, ^19^

Since non‐conveyance practice is integrated into our EMS system, assignment of a control group was not possible for this study and we could not compare mortality between conveyed and non‐conveyed patients. Nearly 90% of all deaths in this dataset occurred in patients >60 years and more than 20% of the deceased were nursing home residents. Physician's telephone consultations were overrepresented in the group of deceased patients, who also more frequently had abnormalities in their vital signs. Collectively these data suggest that the deaths of these patients were expected and that the ambulance crews used all available information to ensure that the terminally ill patient received adequate care. This is further emphasised by the fact that the most important cause of death was a malignancy.

Our results support previous findings of the NEWS being able to predict mortality also in the prehospital setting.^23^ In our study, this also applied to non‐conveyed prehospital patients. The challenge with NEWS scores is that a single numeric score is less informative than its individual components. The risk associated with abnormal respiratory function and level of consciousness was associated with a higher risk than abnormalities in other vital signs. Medical decisions during night hours are often associated with higher risks.^24^, ^25^ The lower mortality during night hours in this study may be explained by the differences in patient material.

The strength of this study is the large, consecutive patient cohort, which was based on uniform electronic prehospital patient reporting. The EPR with automatic transfer of measured vital signs minimised the amount of missing data.

The study was limited by the retrospective study design and a lack of control group for comparing mortality in the EMS system. The study was ambulance crew‐initiated non‐conveyance decisions on adult patients. Thus, patients who had incomplete identity information refused treatment or transport or <16 years were excluded from the study. This means that the patient sample did not represent all non‐conveyance cases in the EMS system and that we could not study other aspects of patient safety beyond mortality. For transported patients, the EMS personnel record a transport code similar to the dispatch code. As the EPR does not enable this for non‐conveyed patients, we were not able to confirm whether the dispatch code was similar to the leading symptom as evaluated by the EMS personnel. DNAR and other relevant treatment limitations were registered as written on the actual prehospital reports, which most likely led to underestimation of their proportion. As all missing vital signs were regarded as normal in this study, it is possible that mortality in the group with no risk factors is lower than that estimated in our study. While we believe the bias to be minimal, this may indicate that the true risk ratios are slightly higher than shown. Patient comorbidities likely play a significant role in the total risk, and the absence of this information limits our ability to compare data with previous studies. A further study that includes comorbidities is required.

## 5 CONCLUSION

Up to one‐third of the patients evaluated by the EMS was not transported to a hospital. All‐cause mortality for non‐conveyed patients was 1.3%. Abnormalities in multiple respiratory‐related vital signs were associated with an increased likelihood of death within 30 days.

## CONFLICT OF INTEREST

The authors have no conflicts of interest.

## Supporting information

Supplementary Material

Supplementary Material
