# Supplementary material for: Ambulance crew‐initiated non‐conveyance in the Helsinki EMS system—A retrospective cohort study
Source: Acta Anaesthesiol Scand. 2022 Feb 28;66(5):625–33. doi: 10.1111/aas.14049 (PMC9544076; doi:10.1111/aas.14049)
Supplement: Supplementary file 1 — Supplementary Material [file AAS-66-625-s001.pdf]

SUMMARY OF GUIDELINES AND EVALUATION OF NON-CONVEYANCE DECISIONS FOR HELSINKI EMERGENCY MEDICAL SERVICES

Summary of non-conveyance guidelines

In all EMS calls in which a patient is not transported to the hospital via ambulance, the reason for non-conveyance is documented for each dispatched unit as follows:

X-0: Technical failure of an ambulance
X-1: Patient died on scene
X-2: Patient taken into police custody
X-3: Other assistance requested on the scene
X-4: Other means of transport (e.g. taxi)
X-5: Neither emergency care nor transport required
X-6: Patients refused transport (against medical advice)
X-7: Patient not found
X-8: Patient treated on the scene
X-9: Ambulance cancelled

In cases where the ambulance is cancelled (X-9) before arriving to the scene or the patient is not found after arriving the scene (X-7), no patient is met. In case of technical failure (X-0), another ambulance is dispatched to complete the EMS call. In case the patient dies on the scene (X-1), the transport of the deceased is handled by a dedicated service.

In all other cases of non-conveyance, where the patient is met by the ambulance crew, the patient should fulfil the following criteria:

1. Reason for the EMS call is clear. The symptoms have passed and the situation does not require further investigation.
2. Patient is able to take care of himself or herself or appropriate supervision is available (e.g. family member, nurse)
3. All appropriate examinations have been made or evaluated to be unnecessary (e.g. minor injury in patient clearly in good condition)
4. No vital parameter is clearly abnormal and an explanation is known for slightly abnormal vital parameters.
5. EMS personnel have evaluated that the patient will not benefit from transport and no other reason supporting patient transport is present.
6. If above criteria are not met or situation remains unclear, EMS personnel are directed to consult the EMS physician.
7. If patient refuses transport (X-6), mental state must be registered to evaluate if patient is competent to make informed decision. In all unclear cases the EMS physician must be consulted to evaluate need for transport against the patient's will.
8. In all elderly nursing home patients, patients with multiple comorbidities or patients with DNAR decisions, evaluation of benefits of transport must be made compared to the care available at present location.
9. If a patient is not transported, the non-conveyance checklist should be filled (see below) and the patient report printed out as written information for the patient.

10.

Non-conveyance checklist found in the electronic patient records (EPR) system

Compulsory for all non-con patients:
Is patient left alone? (Yes/No)
Is patient informed to call 112 again if condition changes? (Y/N)
Is decision of non-conveyance made in mutual agreement with patient? (Y/N)
Is patient able to care of himself or herself in day to day life (Y/N)
Is patient able to walk? (Y/N)
Has patient been given home care instructions?
-what-> (short answer)
Possible follow up instructions given to patient: (short answer)
Other considerations: (short answer)

Additional questions will be included based on the reason for non-conveyance. For example, if a patient refuses transport or is taken to police custody, additional questions focusing on state of mind and possible intoxication will be added.

Implementation into the continuing education system

In addition to this, all guidelines and standard operating procedures on the evaluation and treatment of a patient group include a section concerning non-conveyance criteria within the patient group. Non-conveyance decision making is also included as a part in all educational events focusing on a particular patient group (e.g. evaluation of patient with chest pain)

Quality control of non-conveyance decisions

A portion all EMS calls is selected weekly for closer inspection by EMS physicians and supervising personnel. EPR records are critically evaluated using pre-defined criteria. Factors evaluated include appropriate examination of the patients, adherence to guidelines in patient treatment and medication, correct use of EMS physician consultations, and that transport decisions were performed accordingly and documented properly. In addition to constructive critical feedback, the EMS personnel are also given feedback on EMS calls handled and documented particularly well. The rate of new EMS calls within a 24-hour time period after the non-conveyance decision is monitored as a quality indicator.

EXAMPLES OF POSSIBLE NON-CONVEYANCE CASES
(examples of categories included in this study)

X-2: Patient taken into police custody
Intoxicated has been in a bar fight, with small bumps and bruises. An ambulance arrives to examine the patient and finds him in in good condition with breath ethanol content not excessively high. The police take the patient into custody.
If any medical reason for disorderly behaviour is suspected (e.g. drugs, head trauma, hypoglycaemia) the patient in transported to a hospital.

X-3: Other assistance requested on the scene
Elderly patient with an end stage malignancy is in at-home hospital care. Family members called an ambulance because of deteriorating condition. An ambulance evaluates the situation on-scene and determines that the weakening condition is likely due the malignancy and impeding death. The ambulance evaluates that a trip to the hospital is likely to be a bigger burden to the patient, than any benefits given at the hospital. The ambulance contacts the at-home hospital,who will send a doctor over to evaluate the need for changes in the patient's palliative care. If for example proper pain medication cannot be made available, the patient will be transported.

X-4: Other means of transport
A child falls down and hurts her arm. An ambulance arrives and a splint in placed on the arm. As a bone may possibly broken, a visit to the hospital is required, but as the patient is not in pain, and the mother is present with her car, it is decided that the mother can take the patient to the ED. If patient has abnormalities in vitals, or injuries prevent the use of normal car, the patient will be transported by the ambulance.

X-5: Neither emergency care nor transport required
Patient with a known history of panic attacks get shortness of breath and feels weak and an ambulance is called. The patient calms down when the ambulance arrives. All vitals are normal, and the patient feels that this was similar the previous panic attacks. The patients is discharged on scene.

X-6: Patients refuses transport (against medical advice)
The patient has had weekly epileptic seizures. This time an ambulance is called but the seizure passes, before the ambulance arrives. The patient is feeling well and acts normally and is in a clear state of mind. The ambulance suggests that the patient is taken to the hospital but the patient refuses and there is no indication to transport against the patient's will.

X-8: Patient treated on the scene
A patient with previously known recurrent supraventricular tachycardia (SVT) with a favourable response to intravenous adenosine can be successfully discharged on the scene with advice to contact his or her treating physician. On the other hand, a patient suffering from the first SVT is always transported to the ED regardless of response to adenosine to ensure adequate patient examination, laboratory tests, provision of patient information and follow-up.
