# Supplementary material for: Ambulance crew‐initiated non‐conveyance in the Helsinki EMS system—A retrospective cohort study
Source: Acta Anaesthesiol Scand. 2022 Feb 28;66(5):625–33. doi: 10.1111/aas.14049 (PMC9544076; doi:10.1111/aas.14049)
Supplement: Supplementary file 2 — Supplementary Material [file AAS-66-625-s002.pdf]

ADDITIONAL TABLE 1: Age-adjusted relative risk of mortality for patients with multiple risk factors

Number of risk factors present	Systolic blood pressure	Blood oxygen saturation	Respiratory rate	Level of consciousness	Unable to walk	Shortness of breath	All patients (RR)	Nursing home resident (RR)	General EMS patient (RR)
0							1.0	1.0	1.0
1						x	2.7	2.1	2.8
1					x		3.1	2.9	2.4
1				x			2.7	2.1	2.5
1			x				1.4	2.4	1.1
1		x					2.6	2.0	2.7
1	x						2.5	2.6	2.3
2					x	x	8.3	5.9	6.7
2				x		x	7.1	4.3	6.8
2				x	x		8.2	5.9	5.8
2			x			x	3.9	4.9	3.2
2			x		x		4.5	6.7	2.7
2			x	x			3.9	4.8	2.8
2		x				x	6.9	4.1	7.6
2		x			x		8.0	5.6	6.5
2		x		x			6.9	4.1	6.6
2		x	x				3.7	4.7	3.1
2	x					x	6.5	5.3	6.3
2	x				x		7.5	7.2	5.4
2	x			x			6.5	5.2	5.5
2	x		x				3.5	6.0	2.6
2	x	x					6.3	5.0	6.1
3				x	x	x	20.4	11.4	15.5
3			x		x	x	11.7	12.9	7.6
3			x	x		x	10.1	9.6	7.7
3			x	x	x		11.6	12.7	6.6
3		x			x	x	19.9	11.0	17.1
3		x		x		x	17.3	8.1	17.4
3		x		x	x		19.8	10.9	15.0
3		x	x			x	9.8	9.2	8.6
3		x	x		x		11.3	12.3	7.3
3		x	x	x			9.7	9.1	7.5
3	x				x	x	18.9	13.8	14.5
3	x			x		x	16.4	10.3	14.8
3	x			x	x		18.8	13.6	12.8
3	x		x			x	9.2	11.6	7.2
3	x		x		x		10.7	15.3	6.2
3	x		x	x			9.2	11.5	6.3
3	x	x				x	15.9	9.9	16.3
3	x	x			x		18.3	13.1	14.1
3	x	x		x			15.9	9.8	14.3
3	x	x	x				8.9	11.1	7.0
4			x	x	x	x	27.7	22.5	17.5
4		x		x	x	x	43.3	19.7	36.0
4		x	x		x	x	27.0	21.8	19.2
4		x	x	x		x	23.7	16.9	19.6
4		x	x	x	x		26.9	21.6	17.0
4	x			x	x	x	41.5	23.7	31.3
4	x		x		x	x	25.7	26.0	16.4
4	x		x	x		x	22.5	20.6	16.7
4	x		x	x	x		25.6	25.8	14.4
4	x	x			x	x	40.6	23.0	34.1
4	x	x		x		x	36.1	18.0	34.6
4	x	x		x	x		40.5	22.8	30.5
4	x	x	x			x	22.0	20.0	18.3
4	x	x	x		x		25.0	25.1	15.9
4	x	x	x	x			21.9	19.8	16.2
5		x	x	x	x	x	55.0	34.0	39.8
5	x		x	x	x	x	53.0	38.6	34.8
5	x	x		x	x	x	73.4	35.4	62.6
5	x	x	x		x	x	52.0	37.8	37.7
5	x	x	x	x		x	46.9	31.8	38.3
5	x	x	x	x	x		51.8	37.6	33.9
6	x	x	x	x	x	x	85.8	49.4	67.4

Age dependent risk for all groups is adjusted to median age of whole study population (63.2 years).
Base risk (Relative Risk=1) for each group is the risk of patients with no risk factors.
Relative Risk=1: All patients 0.72%, Nursing home patients 1.46%. General EMS patients 0.69%.
Highest risk combinations within each patient group are highlighted, RR= Relative risk.
